# Sensing and avoiding sick conspecifics requires Gαi2^+^ vomeronasal neurons

**DOI:** 10.1186/s12915-023-01653-8

**Published:** 2023-07-10

**Authors:** Jan Weiss, Hélène Vacher, Anne-Charlotte Trouillet, Trese Leinders-Zufall, Frank Zufall, Pablo Chamero

**Affiliations:** 1 Center for Integrative Physiology and Molecular Medicine, Saarland University, 66421 Homburg, Germany; 2 Laboratoire de Physiologie de la Reproduction et des Comportements, UMR 0085 INRAE-CNRS-IFCE-University of Tours, Nouzilly, France

**Keywords:** LPS, Inflammation, Olfactory, Vomeronasal organ, Gαi2, V1R, Ca^2+^ imaging, Dendritic knob, Avoidance behavior, Lateral habenula

## Abstract

**Background:**

Rodents utilize chemical cues to recognize and avoid other conspecifics infected with pathogens. Infection with pathogens and acute inflammation alter the repertoire and signature of olfactory stimuli emitted by a sick individual. These cues are recognized by healthy conspecifics via the vomeronasal or accessory olfactory system, triggering an innate form of avoidance behavior. However, the molecular identity of the sensory neurons and the higher neural circuits involved in the detection of sick conspecifics remain poorly understood.

**Results:**

We employed mice that are in an acute state of inflammation induced by systemic administration of lipopolysaccharide (LPS). Through conditional knockout of the G-protein Gαi2 and deletion of other key sensory transduction molecules (Trpc2 and a cluster of 16 vomeronasal type 1 receptors), in combination with behavioral testing, subcellular Ca^2+^ imaging, and pS6 and c-Fos neuronal activity mapping in freely behaving mice, we show that the Gαi2^+^ vomeronasal subsystem is required for the detection and avoidance of LPS-treated mice. The active components underlying this avoidance are contained in urine whereas feces extract and two selected bile acids, although detected in a Gαi2-dependent manner, failed to evoke avoidance behavior. Our analyses of dendritic Ca^2+^ responses in vomeronasal sensory neurons provide insight into the discrimination capabilities of these neurons for urine fractions from LPS-treated mice, and how this discrimination depends on Gαi2. We observed Gαi2-dependent stimulation of multiple brain areas including medial amygdala, ventromedial hypothalamus, and periaqueductal grey. We also identified the lateral habenula, a brain region implicated in negative reward prediction in aversive learning, as a previously unknown target involved in these tasks.

**Conclusions:**

Our physiological and behavioral analyses indicate that the sensing and avoidance of LPS-treated sick conspecifics depend on the Gαi2 vomeronasal subsystem. Our observations point to a central role of brain circuits downstream of the olfactory periphery and in the lateral habenula in the detection and avoidance of sick conspecifics, providing new insights into the neural substrates and circuit logic of the sensing of inflammation in mice.

**Supplementary Information:**

The online version contains supplementary material available at 10.1186/s12915-023-01653-8.

## Background

Social behaviors facilitate interactions between conspecifics and increase the likelihood of the transmission of pathogens. Animals have developed behavioral mechanisms to minimize their exposure to infected individuals and to avoid contagion [1, 2]. In particular, rodents distinguish between infected and uninfected conspecifics on the basis of olfactory information, displaying avoidance of infected individuals [1, 3].

Olfactory-mediated social recognition depends largely on the accessory olfactory or vomeronasal system, including the detection of threats such as predator- or pathogen-derived molecules [4–8]. These chemical signals are detected by sensory neurons of the vomeronasal organ (VNO) through members of the vomeronasal type 1 receptor (V1R), vomeronasal type 2 receptor (V2R), and formyl peptide receptor (FPR) families [9, 10]. V1Rs and most FPRs are expressed in the apical VNO layer and use the G protein Gαi2 for signal transduction [11–14], whereas V2Rs and FPR3 employ Gαo signaling in the basal VNO [8, 15]. However, the molecular identity of the sensory neurons in the VNO responsible for the detection of sick conspecifics has remained elusive.

V1Rs are activated by small organic ligands present in urine and feces [14, 16–20], whereas V2Rs are activated by peptides present in urine, fur, or saliva [7, 15, 21, 22]. FPRs were originally identified as innate immune receptors activated by viral and bacterial metabolites and have been implicated in pathogen detection by the VNO [8, 13, 23, 24]. Mice often examine urine and feces and use olfactory cues derived from these sources to distinguish between infected and uninfected individuals. Such odor-driven activity ultimately must be represented and integrated by higher structures in the central nervous system (CNS) to cause active avoidance of infected individuals. Neither the source and chemical nature of the ligands that trigger the detection of sick conspecifics nor the neural circuits mediating avoidance responses have been well described.

We previously investigated chemosensory mechanisms underlying the detection of danger-associated metabolites and the execution of appropriate defense programs, including the sensing of predator cues, life-threatening environmental cues, and bacteria-derived metabolites [8, 25–27]. One important result from these studies was the finding that danger-associated olfactory stimuli causing defensive behaviors such as avoidance can be detected by multiple olfactory subsystems and involve a wide variety of cellular and molecular mechanisms.

Given these results, we here sought to investigate mechanisms underlying the sensing and avoidance of sick conspecifics using mice as experimental model. The first objective of this study was to employ mice that are in an acute state of inflammation induced by systemic administration of lipopolysaccharide (LPS, a component of cell walls of gram-negative bacteria) as a general indicator for the presence of pathogens [3] to gain new insight into the mechanisms mediating the sensing and avoidance of LPS-treated conspecifics. A second goal of this work was to obtain new information on the function of the Gαi2-expressing (Gαi2^+^) subsystem of the VNO. Previous work has shown that this subsystem is involved in the balancing of territorial and infant-directed aggression [14], in the initial outcome of an acute competition [28], and in experience-dependent, VNO-mediated social behaviors [29]. Here, through conditional disruption of Gαi2^+^ and other genes encoding key sensory transduction proteins—in combination with behavioral testing, cellular Ca^2+^ imaging, and pS6 and c-Fos neuronal activity mapping in freely behaving mice—we show that the Gαi2^+^ vomeronasal subsystem also plays a central role in the detection and avoidance of sick conspecifics, and we identify the lateral habenula as a previously unknown CNS target involved in these tasks.

## Results

### Innate avoidance of LPS-treated mice requires Trpc2 and Gαi2 but is independent of the V1rab receptor cluster

**Fig. 1:**
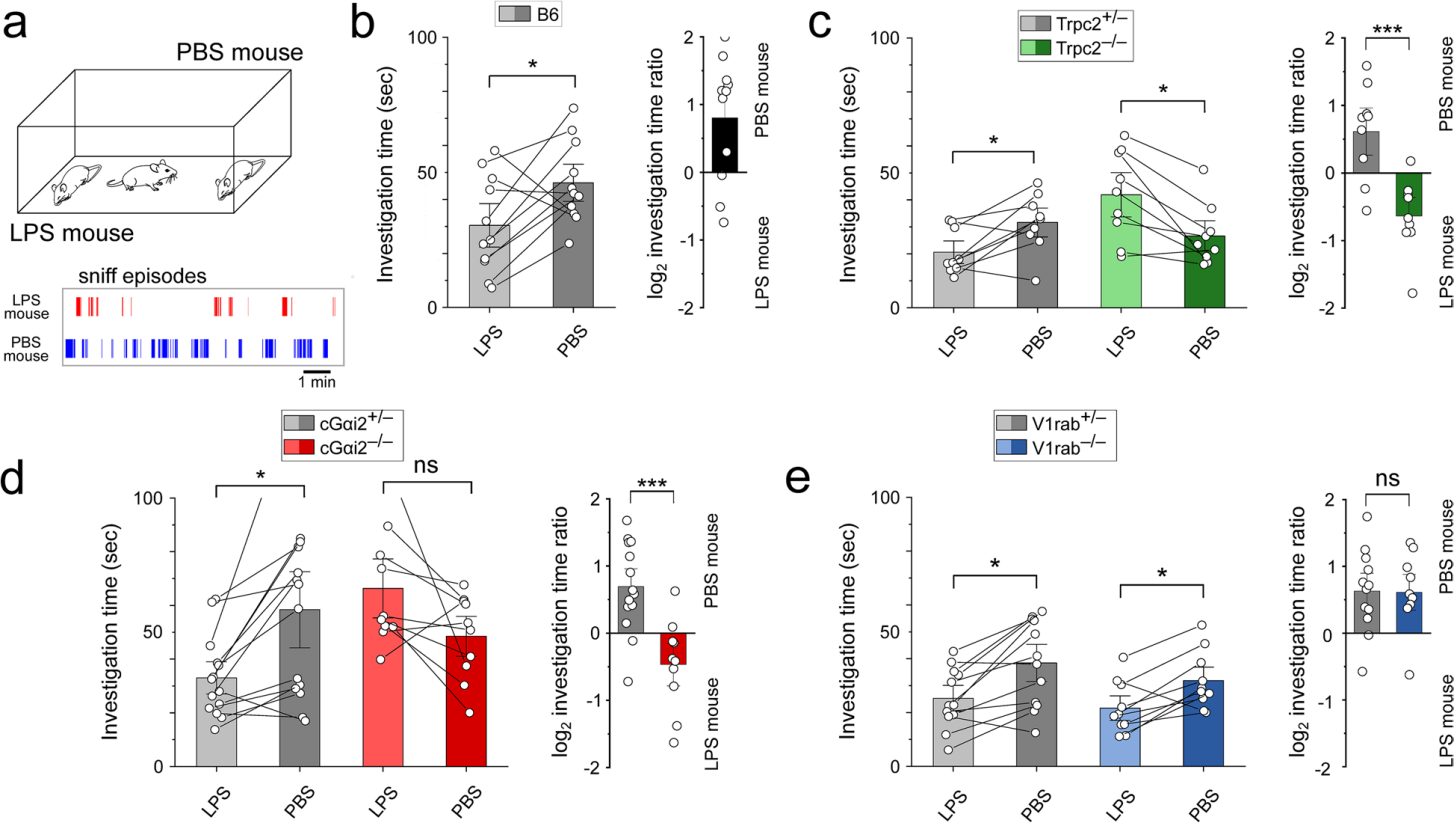
Innate avoidance of LPS-treated mice requires Trpc2 and Gαi2 but is independent of the V1rab receptor cluster. **a** Male mice were allowed to investigate two anesthetized male conspecifics injected with either PBS or LPS. Bottom: representative scoring of the sniff episodes. **b**–**e** Investigation times and log_2_ investigation time ratios (preference score) of PBS/LPS-injected mice for B6, Trpc2^−/−^, cGαi2^−/−^, and V1rab^−/−^ mice and their heterozygous controls. **b** B6 males (mean ± SEM, *n* = 11). **c** Trpc2^+/−^ vs. Trpc2^−/−^ males (*n* = 9, each). **d** Gαi2^+/−^ vs. cGαi2^−/−^ males (*n* = 10, 14, respectively). **e** V1rab^+/−^ vs. V1rab^−/−^ males (*n* = 10, 12, respectively). Unpaired *t*-tests, *p < 0.05, **p < 0.01, ***p < 0.001. ns, not significant *p* = 0.06–0.94. Open circles represent individual mice

Innate avoidance behavior can protect the host from infections and may reduce the spread of pathogens. Mice avoid conspecifics that are in an acute inflammatory state induced by injection of LPS [3], which mimics bacterial infection [30]. The VNO mediates this conspecific avoidance by detecting urinary cues from LPS-injected mice (LPS-urine) [3], but the molecular identity of the vomeronasal sensory neurons (VSNs) sensing these cues has not been characterized further. To identify VSN subpopulations that mediate the avoidance of sick conspecifics, we investigated the behavior of mice harboring three specific deletions of genes that are required for VNO signal transduction (Fig. 1).

First, we focused on the Trpc2 cation channel, the primary sensory ion channel in the VNO [5, 6, 31, 32]. We exposed healthy mice to anesthetized stimulus mice that were injected with either LPS or PBS control solution (Fig. 1a). The LPS-treated mice showed clear signs of sickness with reduced overall activity and an average drop in body temperature of 4.4 °C (LPS-treated: 33.8 ± 0.45 °C, *n* = 17; PBS-treated: 38.1 ± 0.19 °C, *n* = 15; Mann–Whitney ***,* p* < 0.001). We quantified the time that the test mouse investigated a stimulus animal during a 10-min exposure. Control C57BL/6N male mice (referred to as B6) spent more time investigating PBS-injected mice vs. LPS-injected mice (PBS: 46.2 ± 4.4 s, LPS: 30.4 ± 5.1 s, *p* < 0.05) (Fig. 1b). The investigation ratio revealed a clear preference for the healthy mouse (log_2_ investigation time (IT) ratio = 0.81 ± 0.27, one-sample t-test: p < 0.05) (Fig. 1b). Next, we examined mice with a constitutive knockout of *Trpc2* (Trpc2^−/−^) vs. heterozygous control mice (Trpc2^+/−^) [33]. Trpc2^−/−^ mice failed to display a preference for the healthy animal (PBS: 26.6 ± 3.5 s, LPS: 41.9 ± 5.2 s; log_2_ IT ratio = −0.64 ± 0.17), in contrast to their heterozygous littermate controls (PBS: 31.6 ± 3.4 s, LPS: 20.6 ± 2.6 s, *p* < 0.05; log_2_ IT ratio = 0.51 ± 0.22, *p* < 0.001) (Fig. 1c). Thus, Trpc2 is required for the lack of preference or avoidance of LPS-treated mice, results that are consistent with previous observations [3].

Trpc2 not only mediates signal transduction in VSNs but also in subsets of sensory neurons located in the main olfactory epithelium (MOE) [26, 27], making constitutive *Trpc2* knockout mice a much more complicated genetic model than previously anticipated. In fact, *Trpc2* is also required for avoidance behavior triggered by these MOE cells [26, 27]. It was, therefore, necessary to employ an alternative genetic strategy for dissecting innate avoidance of LPS-treated mice. We hypothesized that the LPS-dependent olfactory cues could be of low molecular weight (LMW) and, therefore, focused on the Gαi2-expressing (Gαi2^+^) VSNs. For these experiments, we employed mice carrying an olfactory marker protein (*Omp-Cre*)-driven conditional knockout of Gαi2 (gene name: *Gnai2*) [14, 28, 29]. These mice are referred to as cGαi2 mice.

We found that, closely similar to Trpc2^−/−^ mice, cGαi2^−/−^ mice failed to show a preference for PBS-treated conspecifics, but rather displayed higher investigation times for the LPS-injected animals (Fig. 1d) (cGαi2^+/−^- PBS: 58.4 ± 9.1 s, LPS: 33.0 ± 3.9 s, *p* < 0.05; cGαi2^−/−^- PBS: 48.5 ± 4.7 s, LPS: 66.3 ± 6.9 s, *p* = 0.06; log_2_ IT ratio cGαi2^+/−^ = 0.69 ± 0.18, cGαi2^−/−^- log_2_ IT ratio = −0.47 ± 0.21, *p* < 0.001). Hence, the conditional *Omp-Cre*-driven Gαi2 deletion virtually phenocopied the effects of the global *Trpc2* deletion in the avoidance behavior of LPS-treated conspecifics. These results indicate that Gαi2 and Gαi2^+^ cells are required for mediating the chemosensory responses leading to the avoidance of conspecifics in an acute inflammatory state.

Gαi2^+^ VSNs of the apical layer of the VNE express >240 individual V1 receptor genes, classified into 12 subfamilies (families A-L), as well as 4 formyl peptide receptors (FPRs) [12, 34, 35]. To narrow down the VSN identities potentially involved in the avoidance of sick conspecifics, we next tested mice harboring a cluster gene deletion of 16 V1Rs (V1rab^−/−^) of the A and B families, which represent ~7% of all V1Rs [36]. We found that both V1rab^−/−^ mice and V1rab^+/−^ littermate controls displayed a clear preference for healthy mice (V1rab^+/−^- PBS: 38.4 ± 4.4 s, LPS: 25.3 ± 3.1 s, *p* < 0.05; V1rab^−/−^- PBS: 31.8 ± 3.2 s, LPS: 21.6 ± 2.9 s, *p* < 0.05; preference score: 0.63 ± 0.17 and 0.61 ± 0.17, respectively, *p* = 0.94) (Fig. 1e). Thus, these 16 V1R receptors are not essential in the avoidance of LPS-treated mice.

### LPS-urine drives Gαi2-dependent avoidance and is detected by Gαi2^+^ VSNs in freely behaving mice

**Fig. 2:**
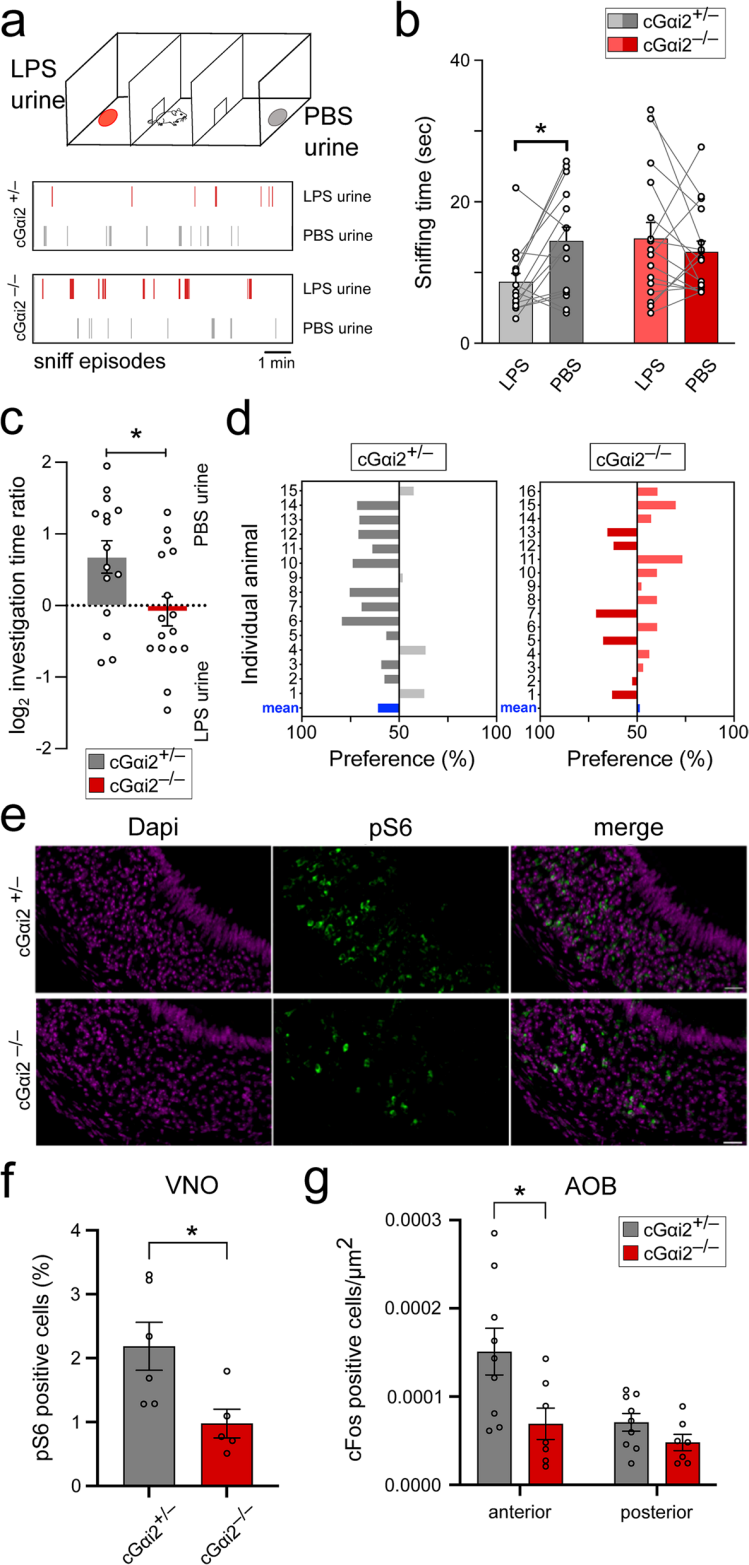
LPS-urine drives Gαi2-dependent avoidance and is detected by Gαi2^+^ VSNs in freely behaving mice.** a** Three-chamber urine investigation assay where male mice could freely investigate filter papers treated with either urine from LPS- (LPS-urine; red) or PBS-treated males (PBS-urine; grey). Filter papers were placed in each lateral compartment. Bottom, examples of sniffing episodes of a control cGαi2^+/−^ mouse and a cGαi2^−/−^ mouse. **b** Investigation times of cGαi2^+/−^ vs. cGαi2^−/−^ mice to LPS-urine and PBS-urine (*n* = 15 and 16 respectively). **c** log_2_ investigation time ratios (preference score) of cGαi2^+/−^ and cGαi2^−/−^ mice to PBS-urine and LPS-urine (*n* = 15 cGαi2^+/−^ and 16 cGαi2^−/−^ mice). **d** Investigation time (preference above 50%) of individual cGαi2^+/−^ and cGαi2^−/−^ mice to PBS-urine or LPS-urine. **e** Representative images of activated VSNs (pS6, green) of cGαi2^+/−^ and cGαi2^−/−^ mice after exposure to LPS-urine. Nuclear DAPI staining in purple. Scale bar: 25 μm. **f** Percentage of pS6 positive cells in VNOs of cGαi2^+/−^ and cGαi2^−/−^ mice after exposure to LPS-urine (n = 6 cGαi2^+/−^ and 5 cGαi2^−/−^ mice). **g** Quantification of c-Fos^+^ cells per μm^2^ in the anterior and posterior AOB of cGαi2^+/−^ and cGαi2^−/−^ mice after exposure to LPS-urine (*n* = 9 cGαi2^+/−^ and 7 cGαi2^−/−^ mice). Mann-Whitney, *p < 0.05. Open circles represent individual mice

LPS-dependent aversive chemosensory cues are present in the urine fraction of LPS-treated mice [3]. We next asked whether the avoidance, or lack of preference, for urine from LPS-treated mice also requires Gαi2. We tested olfactory preference of cGαi2^−/−^ mice vs. cGαi2^+/−^ mice to urine from PBS- or LPS-treated males using a two-choice test. We placed filter papers containing 50 μl of urine from LPS- or PBS-treated males in each lateral compartment of a three-chamber apparatus (Fig. 2a) and analyzed the time spent sniffing each odor source. LPS-urine did not elicit any avoidance in cGαi2^−/−^ mice (PBS: 12.91 ± 1.54 s, LPS: 14.8 ± 2.3 s, *p* = 0.49; preference score: −0.08 ± 0.20), in contrast to cGαi2^+/−^ control animals (PBS: 14.5 ± 1.9 s, LPS: 8.7 ± 1.9 s, *p* < 0.05; preference score: 0.67 ± 0.22,* p* < 0.05) (Fig. 2b, c). Analyses of each mouse showed that the majority (11/15) of cGαi2^+/−^ control animals spent more time investigating PBS-urine, whereas only 6/16 cGαi2^−/−^ mice showed a preference for PBS-urine vs. LPS-urine (Fig. 2d). These results reveal that urine from LPS-treated mice is less attractive in a two-choice test and that the preference for healthy urine requires Gαi2 signaling.

Can LPS-urine activate specific subsets of VSNs in freely behaving mice under in vivo conditions? To address this question, we analyzed VNO activation using immunodetection of the phosphorylated state of the 40S ribosomal protein S6 (pS6) as a proxy of cellular activation [37, 38] after exposing freely moving cGαi2^+/−^ and cGαi2^−/−^ mice to LPS-urine. Exposure to LPS-urine induced pS6 expression in 2.19% of VSNs in cGαi2^+/−^ control mice (Fig. 2e, f). Importantly, the number of pS6-expressing cells was reduced by 55% in the VNO of cGαi2^−/−^ mice after exposure to LPS-urine (0.98%, *p* < 0.05) (Fig. 2f). These results provide evidence that a substantial fraction of LPS-urine is detected by Gαi2^+^ VSNs.

To validate these observations, we also analyzed the expression of the activity-driven c-Fos protein in cells of the accessory olfactory bulb (AOB), the brain structure targeted by VSN axonal projections. Stimulation with LPS-urine induced a significant increase in c-Fos expression in cGαi2^+/−^ but not in cGαi2^−/−^ mice when compared to unstimulated animals (*p* < 0.01). We analyzed the density of c-Fos-positive (c-Fos^+^) cells following LPS-urine exposure in the anteroposterior AOB and observed significantly less c-Fos^+^ cells in the anterior AOB of cGαi2^−/−^ mice vs. cGαi2^+/−^ mice (Fig. 2g; *p* < 0.05). In the posterior AOB, which receives sensory input from Gαo^+^ VSNs, LPS-urine exposure did not induce any significant difference (*p* = 0.21), similar to unstimulated animals (Fig. 2g and Additional file 1: Suppl. Fig. 1). Taken together, these combined results provide strong evidence that exposure to LPS-urine leads to the in vivo activation of the Gαi2^+^ vomeronasal subsystem.

### Selective VSN Ca^2+^ responses to PBS- and LPS-urine require Gαi2

**Fig. 3:**
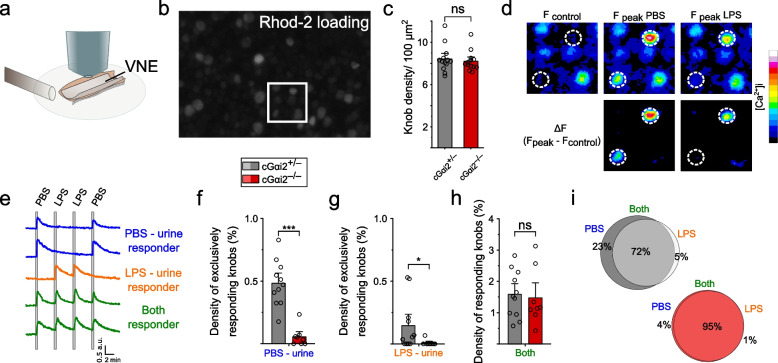
VSN Ca^2+^ responses to LPS-urine require Gαi2. **a** *En face* VNE confocal Ca^2+^ imaging approach. **b** High magnification image of Rhod2/AM loaded vomeronasal knobs (100 × 60 μm). **c** Mean knob density does not differ in cGαi2^+/−^ vs. cGαi2^−/−^ VNOs (*n* = 13, each; Mann-Whitney: ns, *p* = 0.61). **d** Images showing Rhod-2 fluorescence at rest (F_control_), at the peak of the response to male urine from PBS-treated mice (F_peak_ PBS), and at the peak of the response to male urine from LPS-treated mice (F_peak_ LPS). Bottom: ΔF images indicating responsive knobs to PBS- and/or LPS-urine (14 × 14 μm). **e** Example traces of confocal time-lapse recordings in single VSN dendritic knobs showing repeatable responses either to PBS-urine, to LPS-urine, or to both stimuli. **f** Density of knobs that responded to PBS-urine analyzed in control vs. cGαi2^−/−^ mice (11 and 8 recording sites in 6 and 5 animals, respectively; Mann-Whitney, ***p < 0.001). **g** Density of knobs that responded to LPS-urine analyzed in control vs. cGαi2^−/−^ mice (Mann-Whitney, *p < 0.05). **h** Density of knobs that reacted to both PBS- and LPS-urine analyzed in control vs. cGαi2^−/−^ mice (unpaired *t*-test: *t* (17) = 0.29, *p* = 0.77). **i** Venn diagrams indicating the percentages of PBS- and LPS-urine responders and their overlap in control vs. cGαi2^−/−^ VNEs (based on 440 and 272 responding cells, respectively)

We next analyzed the selectivity and discrimination capabilities of native VSNs to LPS-urine vs. PBS-urine using dynamic Ca^2+^ imaging (Fig. 3). For this purpose, we employed *en face* imaging of individual dendritic endings (knobs) using a VNO whole-mount preparation that enables precise visualization of VSN activation patterns in response to chemostimulation [8, 13] and analyzed the density of activated knobs [8] (Fig. 3a). We loaded VSNs of cGαi2^+/−^ and cGαi2^−/−^ mice with the fluorescent Ca^2+^ indicator Rhod-2 and performed time-lapse confocal imaging. The surface of the VNE showed efficient loading of the vast majority of VSN dendritic knobs (Fig. 3b). Knob density was not statistically different between cGαi2^+/−^ and cGαi2^−/−^ VNEs (8.42 ± 0.3 and 8.21 ± 0.3 knobs/100 μm^2^, respectively; *p* = 0.61) (Fig. 3c). Upon application of PBS- and LPS-urine at dilutions of 1:100, we observed synchronized and repeatable intracellular Ca^2+^ transients in numerous well-defined knobs (Fig. 3d). We analyzed 20,239 knobs (see the “Methods” section) in control VNOs and observed three different types of responses: (i) VSNs that were selectively activated by PBS-urine; (ii) VSNs that were selectively activated by LPS-urine; and (iii) VSNs that did not discriminate between both types of stimuli (Fig. 3d, e). Overall, both PBS- and LPS-urine activated a similar number of knobs (PBS-urine: 393 knobs, 2.1 ± 0.14%; LPS-urine: 340 knobs, 1.7 ± 0.25%) (Additional file 1: Suppl. Fig. 2a). These similarities suggest that the majority of knobs detect both stimuli. Indeed, of the responsive VSNs, 72% (1.6% of all knobs) detected both PBS- and LPS-urine, whereas only 23% (0.5% of all knobs) detected only PBS-urine, and 5% (0.1% of all knobs) detected only LPS-urine (Fig. 3f-i).

Next, we analyzed Ca^2+^ responses to PBS- or LPS-urine in cGαi2^−/−^ VNOs by imaging 18,269 individual knobs. There was a striking 10-fold reduction in the number of individual knobs that were activated selectively by PBS- or by LPS-urine (PBS-urine: 0.05 ± 0.03%; *p* < 0.001; LPS-urine: 0.01 ± 0.001%; *p* < 0.05) (Fig. 3f, g). The percentage of responders dropped from 23 to 4% for PBS-urine and from 5 to 1% for LPS-urine in control vs. cGαi2^−/−^ mice in this analysis (Fig. 3i). By contrast, the number of VSNs that detected both PBS- and LPS-urine remained closely similar between genotypes (1.6 ± 0.2% vs. 1.5 ± 0.3% of knobs; *p* = 0.77) (Fig. 3h), suggesting that this type of response is largely independent of Gαi2. Hence, these experiments indicate that conditional deletion of Gαi2 signaling in cGαi2^−/−^ mice leads to a dramatic reduction of cellular responses in those VSNs that are capable of discriminating PBS-urine vs. LPS-urine, and thus indicate that Gαi2 is required for this cellular discrimination.

### Loss of Gαi2 predominantly impairs VSN Ca^2+^ responses to LMW urine fraction from healthy mice

**Fig. 4:**
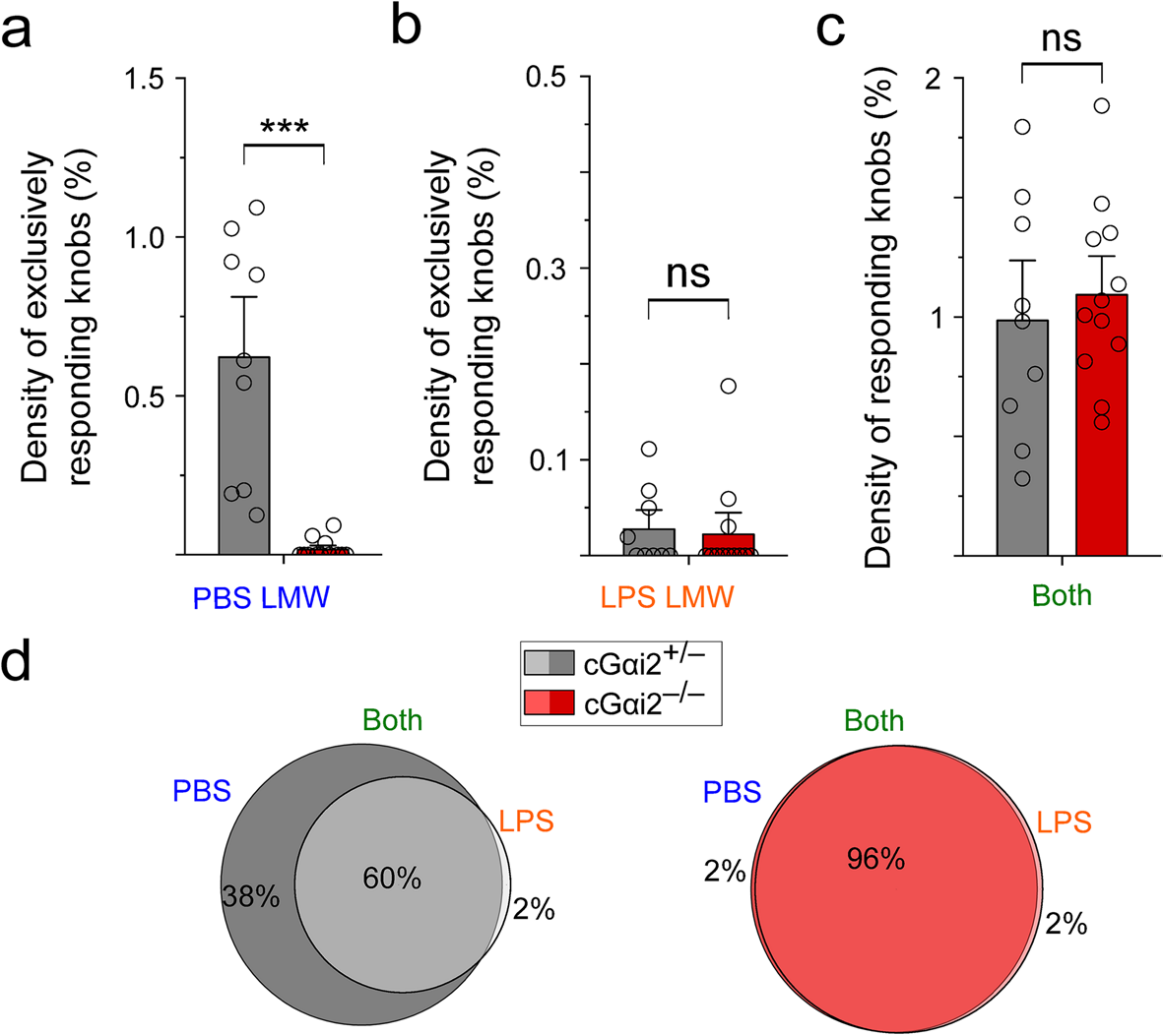
Loss of Gαi2 predominantly impairs VSN Ca^2+^ responses to LMW urine fraction from healthy mice. **a**–**c** Density of knobs that responded to the LMW urine fraction of PBS- or LPS-treated mice analyzed in control vs. cGαi2^−/−^ mice. **a** Density of knobs that responded exclusively to LMW_PBS_ (9 and 12 recording sites in 5 animals each, respectively; Mann-Whitney, ***p < 0.001). **b** Density of knobs that responded exclusively to LMW_LPS_ (Mann-Whitney ns, *p* = 0.44). **c** Density of knobs that responded to both LMW_PBS_ and LMW_LPS_ (unpaired *t*-test: *t* (19) = 0.56, *p* = 0.58). **d** Venn diagrams indicating the percentages of LMW_PBS_ and LMW_LPS_ responders and their overlap in control vs. cGαi2^−/−^ knobs (based on 359 and 312 responding knobs, respectively)

To obtain direct evidence that the main activity of LPS-urine is contained in the LMW urine fraction, we used this LMW fraction (<10 kDa molecular mass) in our VSN Ca^2+^ imaging assay (Fig. 4). Chemicals present in this fraction are primarily detected by Gαi2^+^ VSNs (~70%), but this fraction also contains small peptides and other molecules that are detected by Gαo^+^ VSNs (30%) [14, 39, 40]. We recorded Ca^2+^ responses in 22,261 and 26,943 knobs from control and cGαi2^−/−^ VNOs, respectively, and applied 1:100 dilutions of LMW_PBS_ and LMW_LPS_. Overall, LMW_PBS_ and LMW_LPS_ activated a similar number of dendritic knobs (Additional file 1: Suppl. Fig. 2b), but there was a 33-fold reduction in the number of knobs that responded exclusively to LMW_PBS_ in cGαi2^−/−^ VNOs (0.6 ± 0.12% vs. 0.018%; p < 0.001) (Fig. 4a). This VSN subpopulation represents 38% of LMW-activated knobs in control VNOs but only 2% in cGαi2^−/−^ VNOs (Fig. 4d). By contrast, the number of knobs responding to LMW_LPS_ and those responding to both, LMW_PBS+LPS_, were not significantly different in control vs. cGαi2^−/−^ VNOs (LMW_LPS_: 0.03 ± 0.01% vs. 0.02 ± 0.01%, *p* = 0.44; LMW_PBS+LPS_: 1.00 ± 0.17% vs 1.1 ± 0.12%, *p* = 0.58) (Fig. 4b, c), suggesting that the differences observed with the whole LPS urine preparation (Fig. 3g) originated from molecules >10 kDa. Thus, conditional deletion of Gαi2 signaling predominantly reduced the number of VSNs capable of detecting chemosensory cues present in the LMW urine fraction from healthy mice.

### VSN Ca^2+^ responses to feces extract from healthy and sick mice as well as to bile acids require Gαi2

**Fig. 5:**
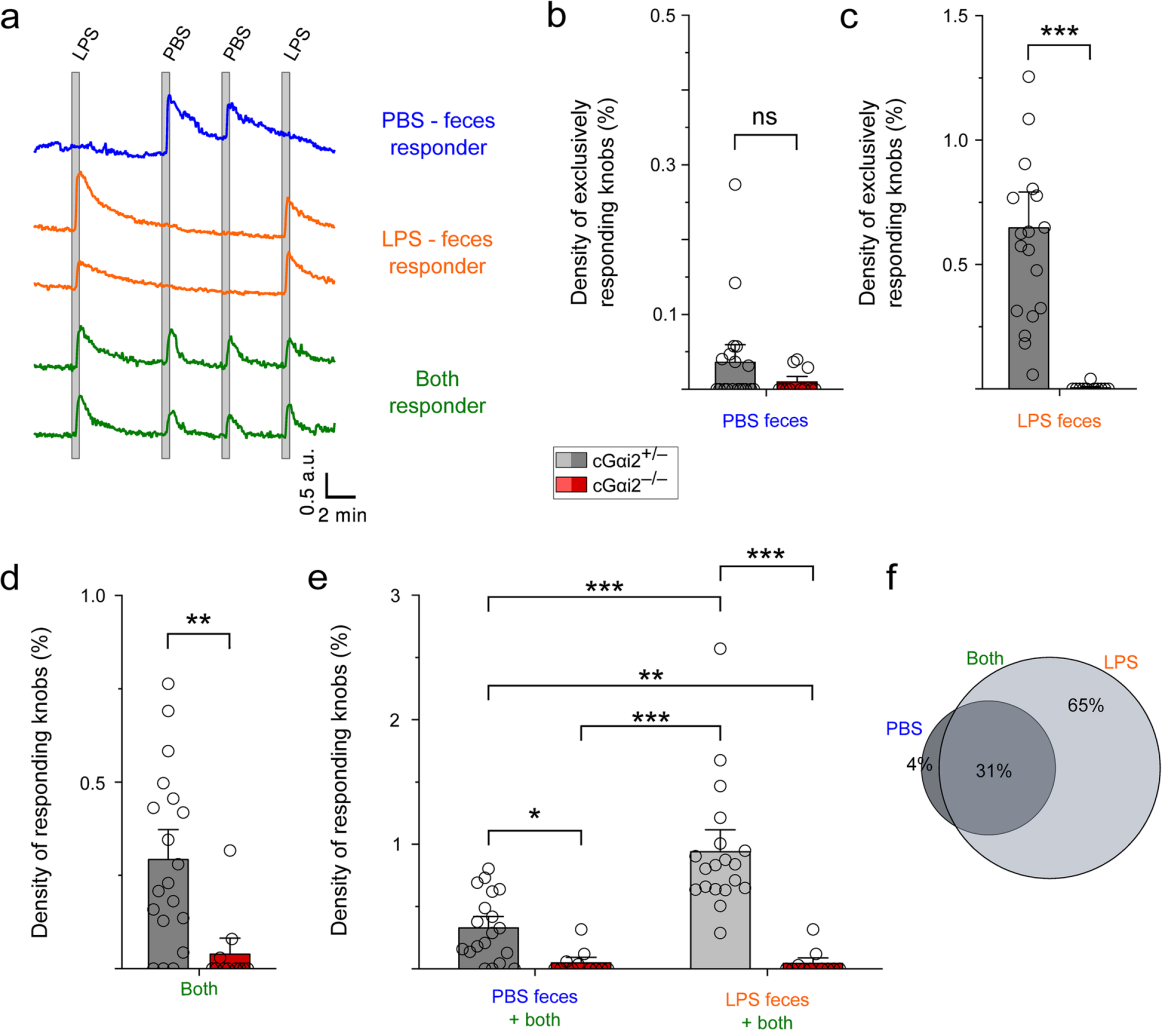
VSN Ca^2+^ responses to feces extract (FE) from LPS- and PBS-treated mice require Gαi2. **a** Example traces of confocal time-lapse recordings in single VSN dendritic knobs showing repeatable responses to PBS-feces, to LPS-feces, or to both stimuli. **b**–**d** Density of knobs responding exclusively to FE solution from either PBS- or LPS-treated mice analyzed in control vs. cGαi2^−/−^ VNEs. **b** Knobs that responded exclusively to PBS-feces in control vs. cGαi2^−/−^ VNEs (19 and 11 recording sites in 10 and 5 animals, respectively; Mann-Whitney ns, *p* = 0.25). **c** Knobs that responded exclusively to LPS-feces (Mann-Whitney, ***p < 0.001). **d** Knobs that responded to both stimuli (Mann-Whitney, **p < 0.01). **e** Density of knobs that responded to PBS- and LPS-feces, either selectively or together (*n* = 19 and 11, respectively; Kruskal–Wallis ANOVA, *p* < 0.001; Mann-Whitney, *p < 0.05; ** p < 0.01; ***p < 0.001). **f** Venn diagram of response percentages to PBS- and LPS-feces in control knobs (based on 444 responding knobs)

Chemical stimuli such as bile acids are found in intestinal luminal contents (feces) and are known to be detected by VSNs [16, 41]. The abundance and nature of such chemicals could be affected in LPS-treated animals as these often show clear signs of diarrhea [42, 43]. We characterized VSN response profiles to feces by analyzing the Ca^2+^ responses of individual VSN dendritic knobs to feces extract (FE, diluted 1:100) from PBS- vs. LPS-injected mice. Similar to stimulation with urine, we found that FE from PBS- or LPS-treated mice could be discriminated by some VSNs whereas other cells reacted to both stimuli (Fig. 5a). In control mice, knobs were primarily activated by LPS-FE (96% of activated knobs), the majority of which (65%) responded exclusively to LPS-FE (Fig. 5c, f). Thus, the density of responding knobs to LPS-FE was 3-fold greater than to PBS-FE (Fig. 5e). Importantly, however, responses to both types of FE were nearly absent in cGαi2^−/−^ VSNs (LPS-FE: 0.6 ± 0.09% vs. 0.004 ± 0.003%; *p* < 0.001; PBS-FE: 0.03 ± 0.02% vs. 0.01 ± 0.004%, *p* = 0.25; PBS-FE plus LPS-FE: 0.3 ± 0.05% vs. 0.036 ± 0.03%; *p* < 0.01) (Fig. 5b–e), indicating that Gαi2 is required for these responses.

We also analyzed VSN Ca^2+^ response profiles to two specific bile acids, cholic acid (CA, 10 μM) and deoxycholic acid (DCA, 10 μM) [16, 41]. Both molecules activated 1.1–1.5% of VSNs in control mice (Additional file 1: Suppl. Fig. 3e), and 75% of FE-sensitive knobs also responded to CA/DCA (Additional file 1: Suppl. Fig. 3f). Here too, Ca^2+^ responses to CA or DCA were virtually absent in VSNs from cGαi2^−/−^ VNOs (Additional file 1: Suppl. Fig. 3b-e). Together, these experiments show that FE from LPS-treated mice evokes altered response profiles in VSNs compared to FE from PBS-treated mice and that these effects are largely Gαi2-dependent. The results also show that VSN responses to two bile acids, CA and DCA, require Gαi2. We, therefore, considered these molecules as candidates to mediate the effects of LPS treatment on avoidance behavior.

### Neither feces extract of LPS-treated mice nor bile acids induce avoidance behavior

**Fig. 6:**
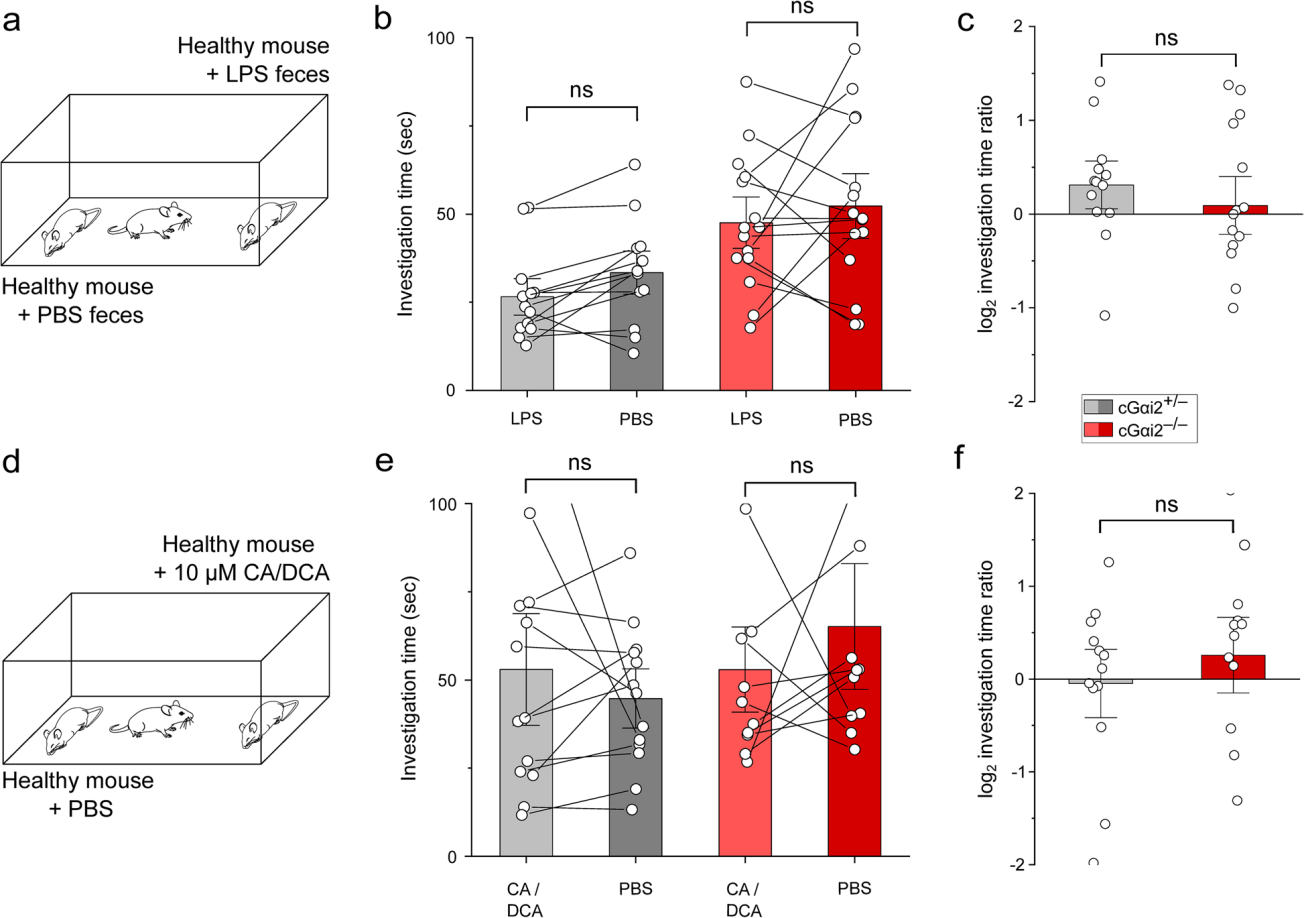
Neither feces extract of LPS-treated mice nor bile acids induce avoidance behavior. **a** Conspecific investigation assay in which male mice were exposed to two healthy anesthetized males painted with FE solution of LPS- or PBS-treated males. **b** Investigation times of cGαi2^+/−^ vs. cGαi2^−/−^ mice (*n* = 13 and 15, respectively; cGαi2^+/−^: unpaired *t*-test: *t* (24) = −1.29, *p* = 0.21, cGαi2^−/−^: unpaired *t*-test: *t* (28) = −0.61, *p* = 0.55). **c** log_2_ investigation time ratios (preference score) (unpaired *t*-test:* t* (26) = −0.8, *p* = 0.43). **d** Male mice were allowed to investigate two healthy anesthetized male conspecifics painted with PBS solution supplemented with 10 μM CA + 10 μM DCA or with PBS alone. **e** Investigation times of healthy conspecifics painted with PBS or with PBS supplemented with CA/DCA in cGαi2^+/−^ vs. cGαi2^−/−^ mice (*n* = 13, 11, respectively; cGαi2^+/−^: Mann-Whitney ns, *p* = 0.76, cGαi2^−/−^: Mann-Whitney ns, *p* = 0.39). **f** log_2_ investigation time ratios (preference score) for healthy conspecifics painted with CA/DCA solution (unpaired *t*-test: *t* (22) = 0.83, *p* = 0.41)

We next tested whether FE from LPS-treated mice, like urine, is sufficient to induce avoidance behavior. We used healthy adult B6 males as stimulus animals and painted their backs and anogenital regions with FE solution from either of two treatments. Stimulus animals were anesthetized and placed in different corners of a test arena (Fig. 6a). cGαi2^+/−^ and cGαi2^−/−^ test males were then introduced and allowed to freely investigate the stimulus animals for 10 min. Unexpectedly, neither cGαi2^+/−^ nor cGαi2^−/−^ males displayed any preference for any of the two conditions (Fig. 6b, c). Furthermore, healthy stimulus B6 mice swabbed with a mixture of two bile acids (CA and DCA, 10 μM each) also did not evoke any preference or avoidance behavior (Fig. 6d–f). Thus, unlike urine and despite being major activators of VSNs, neither FE (from PBS- or LPS-treated mice) nor the bile acids CA and DCA elicited any noticeable preference or avoidance behaviors in our assay. We conclude, therefore, that these types of stimuli can be ruled out in the search for the active compounds mediating inflammation-associated avoidance behavior of sick conspecific mice.

### Information contained in LPS-urine and detected in a Gαi2-dependent manner is represented in multiple brain regions including the lateral habenula

**Fig. 7:**
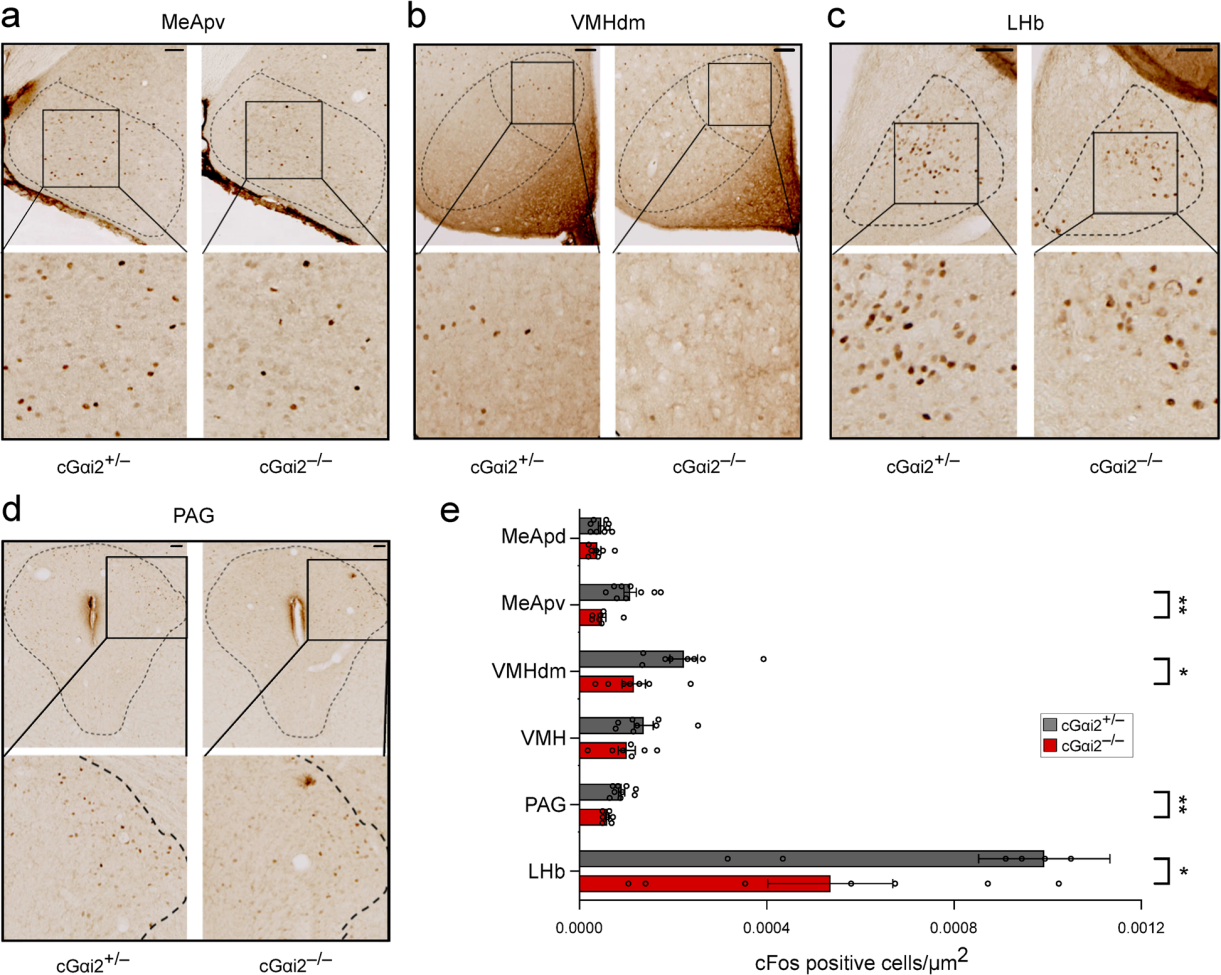
Brain structures activated by LPS-urine. **a** Representative images of c-Fos activation in MeApv, **b** VMHdm, **c** LHb, and **d** PAG (areas delimitated by dashed lines) of cGαi2^+/−^ and cGαi2^−/−^ mice after exposure to LPS-urine. Bottom: higher magnification images of c-Fos^+^ cells. Scale bars: 50 μm. **e** Quantification of c-Fos^+^ cells per μm^2^ in MeApd, MeApv, VMHdm, VMH, PAG, and LHb of cGαi2^+/−^ and cGαi2^−/−^ mice after exposure to LPS-urine. Statistically significant reduction was observed in the MeApv, VMHdm, PAG, and LHb (*n* = 8-9 cGαi2^+/−^ and 7 cGαi2^−/−^ mice). No significant differences were observed in the VMH and MeApd (ns, *p* = 0.33–0.40). Mann-Whitney, *p < 0.05, **p < 0.01. Open circles represent individual mice

Having shown that the representation of LPS-urine is initially processed across the olfactory periphery in the apical layer of VNO and anterior AOB and that these steps occur in a Gαi2-dependent manner (Fig. 2e, f, g), we next asked where in the CNS this information is encoded subsequently. We exposed cGαi2^+/−^ vs. cGαi2^−/−^ males to LPS-urine and analyzed the number of c-Fos^+^ cells in several brain areas including the posterodorsal and posteroventral medial amygdala (MeApd, MeApv), the dorsomedial subdivision of the ventromedial hypothalamus (VMHdm), the periaqueductal grey (PAG), and the lateral habenula (LHb) (Fig. 7 and Additional file 1: Suppl. Fig. 4a). Non-stimulated animals showed equivalent levels of basal c-Fos activity in all studied brain regions (Additional file 1: Suppl. Fig. 4a). Significantly, cGαi2-dependent c-Fos activation after stimulation with LPS-urine occurred in three major vomeronasal target regions: the MeApv, the VMHdm, and PAG (Fig. 7a–e). Notably, c-Fos activity induced by LPS-urine was lower in MeApd, MeApv, and PAG when compared to PBS-urine exposure in control animals (Additional file 1: Suppl. Fig. 4b). Interestingly, we also observed robust, cGαi2-dependent activation in the LHb (Fig. 7c, e), a structure that is activated by primary aversive stimuli [44]. In contrast to other areas, in the LHb, LPS-urine induced stronger c-Fos activation (4-fold increase) when compared to PBS-urine in control animals (Additional file 1: Suppl. Fig. 4c). Other areas of the medial amygdala and ventromedial hypothalamus lacked significant effects of the disruption of Gαi2 signaling (Fig. 7e).

Together, these results indicate that inflammation-associated odor information contained in conspecific LPS-urine must ultimately target and engage the MeApv, VMHdm, PAG, and LHb, and requires intact Gαi2 vomeronasal function.

## Discussion

This study provides new insights into the full range of cellular and molecular parameters underlying the sensing and avoiding of sick conspecifics in mice. As such, this work confirms and extends a previous investigation on this topic [3]. Several new results emerge from our work: (1) The demonstration that the G protein Gαi2 and the Gαi2^+^ population of VSNs are required for the sensing and avoidance of conspecific mice that are in an acute state of inflammation; (2) the demonstration that conditional deletion of Gαi2 in the olfactory system phenocopies the effect of a constitutive *Trpc2* knockout with regard to sick conspecific avoidance, and that a cluster deletion of 16 V1Rs has no impact on avoidance of LPS-treated conspecifics; (3) the finding that the active components underlying the sensing of LPS-injected mice are contained in the urine fraction, but not in feces; (4) the result that two selected bile acids require Gαi2 for VSN activation but do not mimic the effects of LPS-urine on avoidance behavior; (5) the detailed analysis of dynamic Ca^2+^ responses in individual VSN dendritic knobs, that these cells can discriminate LPS- and PBS-urine and its LMW fraction, and how this discrimination depends on Gαi2; (6) the demonstration that the sensing and avoidance of sick conspecifics engages, in a Gαi2-dependent manner, the activation of multiple brain areas including the medial amygdala, the ventromedial hypothalamus, the periaqueductal grey, and the lateral habenula.

Rodents display a variety of behaviors and strategies, including chemosensation mediated by the accessory olfactory system, in order to avoid conspecifics that show signs of pathogen infection [3, 45, 46]. Our results demonstrate that the Gαi2^+^ population of VSNs controls important features of sick conspecific discrimination. Specifically, we found that conditional deletion of Gαi2 suppresses preference behavior for healthy mice, consistent with a direct role of Gαi2 in the detection of an acute inflammatory state in conspecifics induced by LPS injection. Apical Gαi2 VSN subpopulations express ~240 V1Rs and 4 FPRs [35]. The precise identities of the specific receptors involved in the detection of chemosensory cues from sick conspecifics are unknown and remain to be investigated in future work, but our results employing a mouse line with a V1R cluster deletion [36] at least exclude the involvement of the type A and B subfamilies of V1Rs.

Excretions like urine and feces have been proposed as potential sources of odors specific to sick individuals [3, 45, 47]. Consistent with this, we observed that urine from LPS-injected mice is less attractive to healthy animals and that this preference requires Gαi2 signaling. Furthermore, analysis of sensory activity in the VSN dendritic knob layer shows that responses specific to urine from sick or healthy animals are preferentially detected by Gαi2^+^ VSNs. This result is consistent with metabolomic characterizations of urine samples in states of inflammation that reported increases as well as decreases in volatile compounds, including V1R-specific ligands [48, 49]. Thus, the lack of preference for urine from sick animals may be caused by either a decrease in attractive olfactory signals or by the presence of repulsive cues in the urine. In this context, we have observed a greater number of specific VSN responses and increased c-Fos activity in the MeA and PAG in response to urine from healthy animals, indicating that an acute inflammatory state could lead to a loss or reduction of vomeronasal signaling. Moreover, specific responses to LMW urine fraction of healthy animals were strongly reduced in Gαi2 mutants, suggesting a possible role of the decrease of attractive cues after LPS injection. Further research will be needed to validate these possibilities. Importantly, we observed that application of LPS-feces extract nearly tripled the number of stimulated VSNs, indicating that treatment with LPS resulted in an increase of VNO ligands in feces, likely through changes in bile acid metabolism [50, 51]. Indeed, we identified two bile acids, CA and DCA, whose activity added up to approximately 75% of the feces-sensitive VSNs in a Gαi2-dependent manner. Although this is consistent with previous results linking the detection of feces and bile acids to V1Rs [16, 41], neither feces extract (LPS- or PBS-treated) nor the bile acids tested here were sufficient to induce an avoidance behavior, leading us to conclude that neither of these stimuli plays a critical role in the sensing and avoidance of LPS-treated mice.

Our c-Fos mapping results indicate that the representation of LPS-urine in the CNS involves several downstream brain areas of the Gαi2 vomeronasal pathway: AOB, MeApv, VMHdm, and PAG. These results are consistent with a number of studies implicating these brain areas in defensive, avoidance, and escape behaviors, including avoidance to predator odors [25, 52–55]. Remarkably, stimulation with LPS-urine induced fewer c-Fos^+^ cells in MeA and PAG as compared with PBS-urine in control animals, which is consistent with the higher sensory activity observed in VSN dendritic knobs in response to specific PBS- vs LPS-urine signals. By contrast, we observed greater Gαi2-dependent c-Fos activity by LPS-urine in the LHb, a phylogenetically ancient brain region that is activated by aversive stimuli and modulates conspecific interactions [44, 56]. The LHb receives inputs from limbic structures and targets midbrain neuromodulatory systems, such as the dopaminergic and serotonergic systems, underlying negative emotional states and negative reward [57]. To the best of our knowledge, these are the first results to implicate the lateral habenula in the sensing and avoidance of chemosensory cues associated with conspecifics that are in acute inflammatory state.

## Conclusions

In summary, our results indicate that the sensing and avoidance of LPS-treated sick conspecifics critically depend on the Gαi2^+^ vomeronasal subsystem. In particular, our observations point to a central role of brain circuits downstream of the olfactory periphery in the AOB, MeApv, VMHdm, PAG, and LHb in the control of sick conspecific avoidance. Our results are consistent with studies implicating the MePV and VMHdm in defensive, escape, and avoidance behaviors and the LHb in negative reward prediction in aversive learning. By identifying specific molecular properties of the sensory neurons that mediate sick conspecific discrimination, we have provided new insights into the genetic substrates and circuit logic of the sensing of inflammation in mice. These results should facilitate further studies aimed at understanding the active chemicals, their receptors, and the neural circuits underlying the perception of sickness by conspecifics.

## Methods

### Mice

Experiments were performed on adult, 8–20-week-old male mice. We employed the following genotypes: (1) Wild-type mice (C57BL/6N, denoted as B6) were obtained from Charles River Laboratories (Sulzfeld, Germany). (2) Mice harboring a targeted, global deletion of the *Trpc2* gene (B6;129P2-Trpc2 < tm2Mom > /MomJ, Stock# 006733; RRID:IMSR_JAX:006733; denoted as Trpc2^−/−^ mice) [33]. (3) Conditional Gαi2 knockout mice (denoted as cGαi2^−/−^) harboring a Cre recombinase-mediated ablation of the *Gnai2* gene under the control of the olfactory marker protein (*Omp*) promoter and generated as described [14]. Mice were homozygous for the floxed *Gnai2* alleles and heterozygous for Cre and *Omp* (*Gnai2*^fx/fx^ *Omp*^cre/+^ or cGαi2^−/−^). In these mice, Cre-mediated *Gnai2* deletion was restricted to *Omp*-positive cells. Animals heterozygous for both alleles (*Gnai2*^fx/+^ *Omp*^cre/+^ or cGαi2^+/−^) or homozygous *Gnai2*^+/+^ *Omp*^+/+^ (cGαi2^+/+^) served as controls. (4) Mice harboring a targeted, global deletion of 16 intact V1r genes of families A and B (129S-Del(6)1Mom/MomJ, RRID:IMSR_JAX:006653, common name ΔV1rabΔ, denoted as V1rab^−/−^ [36]. Control mice were heterozygous littermates. Mice were housed in individually ventilated cages (IVCs) on a 12:12-h light-dark cycle with food and water available *ad libitum*.

### Treatment of mice with LPS and urine and feces collection

Male mice (B6, 8–20 weeks old) were injected intraperitoneally with 1–5 mg/kg LPS (Lipopolysaccharide L4516 and L4391, Sigma, in 200 μl phosphate-buffered saline, PBS) depending on the endotoxin units (EU) according to the specification sheet of the Lot (3,000,000 EU/kg). Control mice received 200 μl PBS injections. Mice were returned into their individual home cages and were used as stimulus animals 4 h post-injection. Urine and feces from several mice injected with LPS or PBS were collected (4 h post-injection), pooled and stored at −80 °C until use. To obtain urine fractions, 0.5 ml of PBS- or LPS-urine was size-fractionated by centrifugation (14,000 × g for 30 min) using Nanosep (Pall) 10-kDa molecular mass cutoff ultrafiltration columns. The centrifugation supernatant was the LMW fraction (<10 kDa). Feces was diluted 1:10 (w:v) in water, vortexed, and left overnight on a shaker at 4° C. The suspension was then centrifuged two times at 2400×*g* for 20 min. The supernatant was aliquoted and stored at −80 °C until use. To measure rectal temperature of the injected animals, mice were placed on a horizontal surface, e.g., a cage lid. The tail was then lifted, and a probe of a fast-acquiring thermometer (<1 s, DTM light, LKM electronic, Geraberg) was gently inserted into the rectum.

### VNO whole-mount preparation and *en face* Ca^2+^ imaging

Mice were deeply anesthetized with CO_2_, sacrificed by decapitation, and VNOs were rapidly removed and dissected in ice-cold oxygenated (95% O_2_, 5% CO_2_) S1 solution containing (in mM) 120 NaCl, 25 NaHCO_3_, 5 KCl, 5 N,N-bis(2-hydroxyethyl)-2-aminoethanesulfonic acid (BES), 1 MgSO_4_, 1 CaCl_2_, 10 glucose, and pH 7.3 (osmolarity: 300 mOsm/l). One half of the VNO within the bony capsule was glued (Loctite 401) to a petri dish (Ø 4 cm), the bony capsule was opened with fine forceps, and the non-sensory tissue was stripped off to expose the VNE to gain access to the surface of the VSN dendritic knobs. Tissue and cell debris as well as the posterior vomeronasal glands were removed and the *en face* preparation was then loaded with Rhod-2/AM (15 μM) calcium dye. Loading was performed at room temperature in carbogenated S1 solution for 1 h. Rhod-2 solution was then removed, and the petri dish was placed on an upright confocal laser scanning microscope (Leica TCS SP5 II, 20× water immersion objective HCX APO L20×/1.0w) equipped with Ar and He/Ne lasers. Rhod-2 was excited at 543 nm, emission was measured between 560 and 680 nm. Images were collected every 1.5 s (1024 × 1024). Stimuli were applied to the VNO surface using a local perfusion system which produced a continuous solution stream (Fig. 3a). All stimuli were diluted in S1 solution and applied for 30 s at least twice during an experiment. Interstimulus interval was at least 4 min. We used urine (1:100 dilution), LMW fraction urine (1:100), and feces solution (1:100) from PBS- or LPS-injected B6 males (see the “Treatment of mice with LPS and urine and feces collection” section). Stock solutions of cholic acid (CA) and deoxycholic acid (DCA) were prepared in methanol and ethanol, respectively, and stored at 4° C. Final solutions (10 μM each) were prepared immediately before use in oxygenated S1 solution. All physiological measurements were performed at room temperature. Data analysis was performed with ImageJ (NIH). Time series stacks were aligned using the Template Matching plugin. Ca^2+^ responses of individual dendritic knobs were normalized to the knob resting fluorescence level obtained before stimulation (ratio Fx/F0, Fx = actual fluorescence; F0 = mean fluorescence of the first 50 images of the experiment). The following criteria for stimulus-induced Ca^2+^ responses were applied. (i) A response was defined as a stimulus-dependent deviation of fluorescence that exceeded twice the SD of the mean of the baseline fluorescence noise. (ii) A response had to occur within 1 min after stimulus application. (iii) Knobs were considered responsive if they reacted to a given stimulus during both applications. For calculation of VSN knob density, we analyzed randomly selected VNO regions (40 × 40 μm) in 13 recorded areas of cGαi2^+/−^ and cGαi2^−/−^ VNOs and counted knob-like structures. The mean density was 8.42 ± 0.35 and 8.21 ± 0.26 knobs/100 μm^2^, respectively. The mean density of all analyzed areas (cGαi2^+/−^ and cGαi2^−/−^) of 8.32 knobs/100 μm^2^ was used to calculate the total knob number in the recorded area. Sampling areas comprised ~13,000 to ~ 40,000 μm^2^ per experiment leading to calculated knob numbers of ~1100 to ~3300 knobs. The number of responding knobs was then quantified in relation to sample area and thus calculated total knob number [8].

### Behavioral testing

All behavioral tests were conducted with adult (8–20-week-old) test and stimulus animals. Habituation and tests were always conducted during the dark phase in a behavior room under infrared light conditions (21 °C, humidity >40%). Experiments were digitally recorded and subsequently analyzed by a blind experimenter.

### Conspecific investigation

We tested preference by giving adult male mice of various genotypes a choice to freely investigate two types of stimulus animals: PBS- vs. LPS-injected anesthetized B6 mice. Test mice were habituated for 2–3 days (for 10 min each day) by introducing cage mates (2–3 mice /cage) into an empty type II cage (floor area 32 × 16 cm) that was secured by a Plexiglas attachment. After 10 min, mice were returned to their home cages. On testing days, stimulus mice (4 h post-injection with either LPS or PBS) were anesthetized with 100 mg/kg ketamine (Serumwerk Bernburg, Bernburg, Germany) and 8 mg/kg xylazine (Serumwerk Bernburg, Bernburg, Germany) and placed on opposite sides of a neutral empty cage. We also tested the preference by adult male cGαi2 mice to freely investigate two types of stimulus mice: (1) healthy anesthetized B6 mice painted with 70 μl feces solution of LPS- or PBS-injected stimulus mice at the anogenital region and back; (2) healthy anesthetized B6 mice painted with PBS solution supplemented with CA/DCA (10 μM each) or PBS solution alone (70 μl) at the anogenital region and back. The test mouse was placed between the two stimulus mice and recorded for 10 min with an infrared digital video camera. All mice showed a keen interest in investigating the stimulus source during which their nose was in close contact with the stimulus animals. Investigative behavior was scored manually using Behavioral Observation Research Interactive Software (BORIS) [58]. Stimulus mice were used in several consecutive trials. We did not observe any influence of trial number on investigation times. The avoidance index was calculated as log_2_ investigation time ratio = log_2_ IT_PBS_/IT_LPS_, with IT_PBS_ as the time a mouse investigated the PBS stimulus animal and IT_LPS_ as the time a mouse investigated the LPS stimulus mouse. Negative values represent preference for the LPS-treated stimulus animal and positive values represent preference for the PBS-treated stimulus animal.

### Urine investigation assay

Two days before a test, mice were daily habituated to the three chambers box for 10 min with unscented stimuli in each side chamber. On the test day, 50 μl of urine from LPS-treated and PBS-treated males were placed on filter papers in each lateral compartment. The sides containing the stimuli were randomized, and the apparatus was cleaned with 20% ethanol between subjects. Mice were free to investigate the apparatus for 10 min. The time spent in each chamber and the duration of chemosensory investigation were scored and an avoidance index calculated as described above.

### Immunostaining

#### Tissue preparation

Mice were individually housed for at least 4 days and exposed to either a clean filter paper, or a filter paper with 50 μl of LPS-treated male urine. Ninety minutes after continuous exposure, mice were anesthetized by an overdose of pentobarbital (Ceva) and perfused transcardially with 0.9% saline solution followed by 0.1 M phosphate buffer (PB) containing 4% paraformaldehyde (PFA). Brains and VNOs were removed, postfixed overnight in 4% PFA, and cryoprotected in 0.1 M PB containing 30% sucrose. Brains, VNOs and olfactory bulbs (OB) were embedded separately in Tissue-Tek® O.C.T™ compound, snap-frozen in cold isopentane, and processed on a Leica CM 3050S cryostat. Brain samples were cut in 30-μm serial free-floating sections (coronal for brains, sagittal for OB) using tris-buffered saline solution (TBS) containing 0.1% sodium azide. VNOs were cut in 16-μm serial coronal sections and directly mounted on SuperFrost Plus slides.

#### pS6 immunolabeling

Slides were washed (3 × 5 min) in TBS, incubated in blocking solution (TBS containing 0.3% Triton X-100, TBS-T, and 2% donkey serum) 2 h at room temperature (RT), and overnight at 4 °C in blocking solution supplemented with the pS6 primary antibody (1:2500; rabbit polyclonal #44-923G, Invitrogen). Slides were then washed in TBS and incubated in TBS-T supplemented with secondary antibody (1:1000 Cy3-conjugated donkey anti-rabbit IgG, Jackson ImmunoResearch) for 2 h at RT. Nuclei were counterstained 5 min at RT with DAPI. Slides were mounted with Fluoromount-G™ (Invitrogen).

#### c-Fos immunolabeling

Sections were washed (3 × 5 min) in TBS; endogenous peroxidases were blocked for 30 min in TBS containing 3% H_2_O_2_. Sections were incubated in blocking solution (TBS containing 0.1% Triton X-100, TBS-T, and 5% normal goat serum) 2 h at RT, and then overnight at 4 °C in blocking solution supplemented with the c-Fos primary antibody (1:1000 mouse monoclonal #sc271243, Santa Cruz Biotechnology). Sections were then washed in TBS and incubated in TBS-T supplemented with secondary antibody (1:1000; biotinylated goat anti-mouse IgG, Jackson ImmunoResearch) for 2 h at RT. Signals were amplified with VECTASTAIN® ABC kit (Vector) for 1 h at RT and then visualized with diaminobenzidine (DAB 0,02%, 0,01% H_2_O_2_ in 0,05 M Tris, pH 7,4). Slides were mounted with DPX (Sigma-Aldrich).

#### Analysis

For c-Fos experiments, slides were scanned using an automatic slide scanner (Axio Scan.Z1, Zeiss). Regions of interest were drawn from scanned brain sections based on the Paxinos mouse brain atlas using QuPath tools [59]. Coronal sections were selected for the LHb, 3–5 sections between bregma −1.22 mm and −1.70 mm; for MeA and VMH, 3–5 sections between bregma −1.34 mm and −1.70 mm; and for PAG, 3–6 sections between bregma −3.40 mm and −3.88 mm. The number of c-Fos^+^ nuclei in each drawn region was automatically counted using the built-in Cell Detection method. The same cell detection parameters, such as set up, nucleus, and intensity parameters, were applied to all the regions. pS6-positive cells were counted using Zen software (blue edition 3.0, Zeiss) and the particle analyzer plug-in of Fiji [60] on 6–15 VNO images per animal and expressed as a proportion of the total DAPI-positive nuclei. For both c-Fos and pS6 measurements, a manual validation of all positive detected cells was performed before exporting all values.

### Statistics

Statistical analyses were performed using the software Origin Pro 2021 (OriginLab Corporation, Northampton, MA, USA) and GraphPad Prism 9.0 (GraphPad Software). Assumptions of normality were tested before conducting the following statistical tests. Student’s *t*-test was used to measure the significance of the differences between two distributions. In case the results failed the test of normality, Mann-Whitney test was performed. Multiple groups were compared using Kruskal-Wallis one-way analysis of variance (ANOVA) with Mann-Whitney and Holm-Šídák’s tests as a post hoc comparison. The probability of error level (alpha) was chosen to be 0.05. The statistical tests used were two-sided. Unless otherwise stated, data are expressed as mean ± standard error of the mean (SEM). Specific statistics and number of samples analyzed are described in the figure captions.

## Supplementary Information

**Additional file 1:** **Suppl. Fig. 1.** No significant difference in c-Fos activation in anterior and posterior AOB of non-stimulated cGαi2^+/^^−^ and cGαi2^−/−^ mice. **Suppl. Fig. 2.** Overall, both PBS-and LPS-urine as well as LMW urine fractions activated a similar number of VSN dendritic knobs. **Suppl. Fig. 3.** VSN Ca^2+^ responses to two selected bile acids (CA and DCA) require Gαi2. **Suppl. Fig. 4.** No significant difference in c-Fos activation in brain regions of non-stimulated cGαi2^+/^^−^ and cGαi2^−/−^ mice.

**Additional file 2:** **Dataset S1.** Dataset containing values andstatistical analyses displayed in all the figures.

## Data Availability

All data generated or analyzed during this study are included in this published article and its supplementary information files. Individual data values are provided in Additional File 2.

## Abbreviations

AOB: Accessory olfactory bulb
CA: Cholic acid
CNS: Central nervous system
DCA: Deoxycholic acid
FE: Feces extract
FPR: Formyl peptide receptor
IT: Investigation time
LHb: Lateral habenula
LMW: Low molecular weight
LPS: Lipopolysaccharide
MeApd: Posterodorsal medial amygdala
MeApv: Posteroventral medial amygdala
MOE: Main olfactory epithelium
OMP: Olfactory marker protein
PAG: Periaqueductal grey
PBS: Phosphate-buffered saline
pS6: 40S ribosomal protein S6
V1R: Vomeronasal type 1 receptor
V2R: Vomeronasal type 2 receptor
VMH: Ventromedial hypothalamus
VMHdm: Dorsomedial subdivision of the ventromedial hypothalamus
VNO: Vomeronasal organ
VSNs: Vomeronasal sensory neurons
