# Supplementary material for: Sensing and avoiding sick conspecifics requires Gαi2+ vomeronasal neurons
Source: BMC Biol. 2023 Jul 10;21:152. doi: 10.1186/s12915-023-01653-8 (PMC10332101; doi:10.1186/s12915-023-01653-8)
Supplement: Supplementary file 1 — Additional file 1: Suppl. Fig. 1. No significant difference in c-Fos activation in anterior and posterior AOB of non-stimulated cGαi2+/− and cGαi2−/− mice. Suppl. Fig. 2. Overall, both PBS-and LPS-urine as well as LMW urine fractions activated a similar number of VSN dendritic knobs. Suppl. Fig. 3. VSN Ca2+ responses to two selected bile acids (CA and DCA) require Gαi2. Suppl. Fig. 4. No significant difference in c-Fos activation in brain regions of non-stimulated cGαi2+/− and cGαi2−/− mice. [file 12915_2023_1653_MOESM1_ESM.pdf]

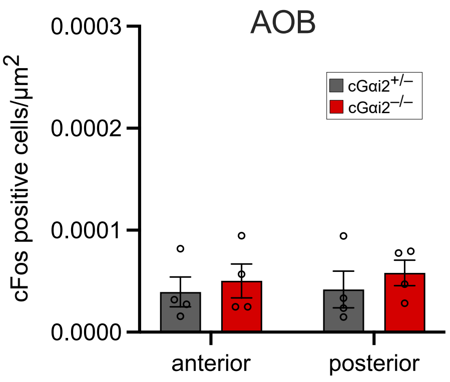

Suppl. Fig. 1. No significant difference in c-Fos activation in anterior and posterior AOB of non-stimulated cGαi2$^{+/–}$ and cGαi2$^{–/–}$ mice. Quantification of c-Fos^{+} cells per µm^{2} in anterior and posterior AOB of cGαi2$^{+/–}$ and cGαi2$^{–/–}$ unexposed mice (n = 4 respectively, Mann-Whitney p= 0.48-0.88).

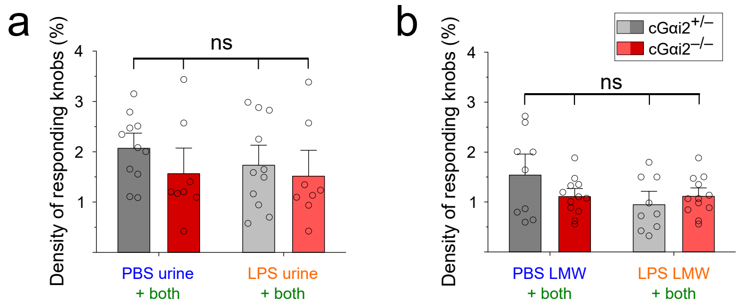

Suppl. Fig. 2. Overall, both PBS- and LPS-urine as well as LMW urine fractions activated a similar number of VSN dendritic knobs. (**a**) Density of responding knobs in VNEs of control vs. cGαi2$^{-/-}$ mice to urine of PBS- or LPS-treated mice (ANOVA $F\ (3, 38) = 0.83$; $p = 0.48$). (**b**) Density of responding knobs in VNEs of control vs. cGαi2$^{-/-}$ mice to LMW urine fraction obtained from PBS- or LPS-treated mice (ANOVA $F\ (3, 38) = 2.02$; $p = 0.13$).

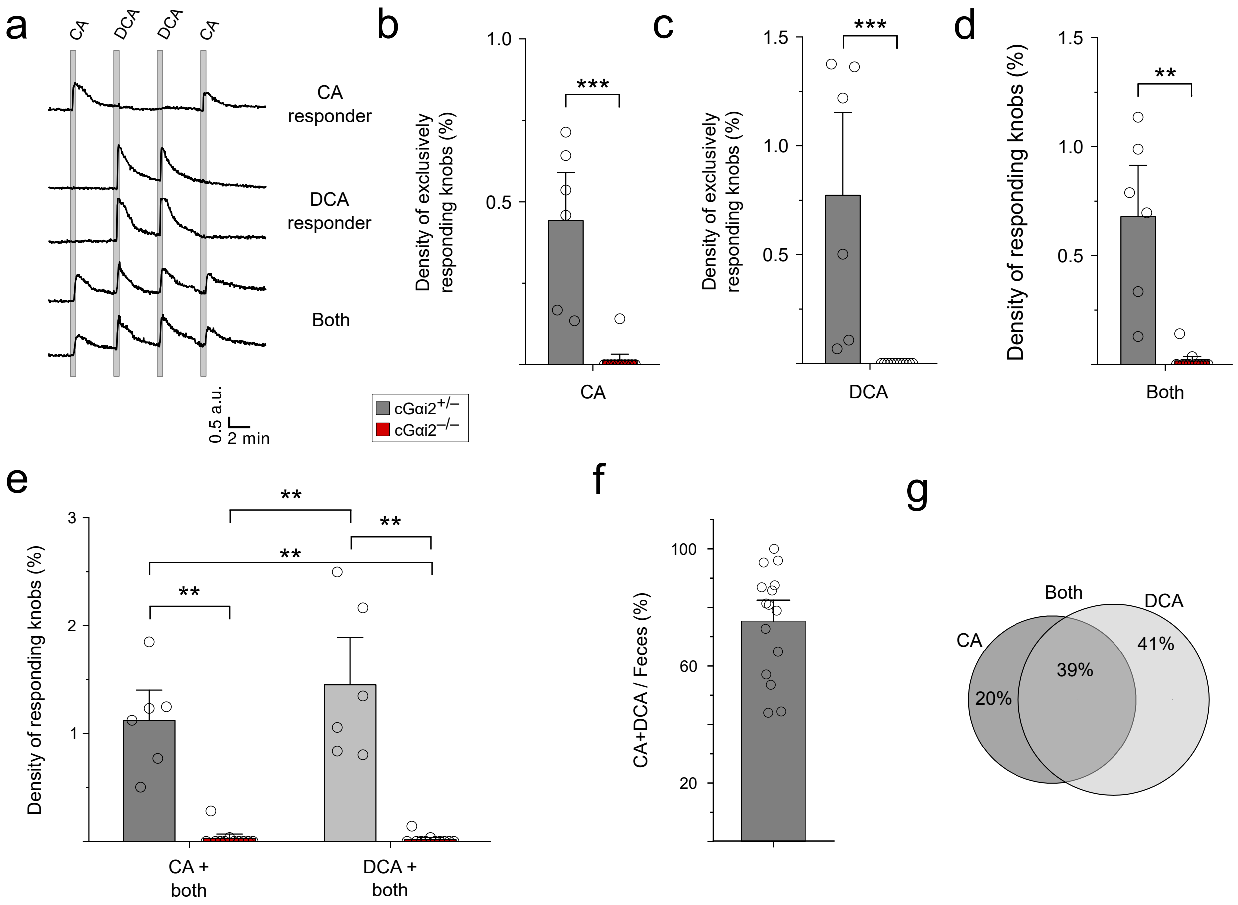

Suppl. Fig. 3. VSN Ca^{2+} responses to two selected bile acids (CA and DCA) require Gαi2. (**a**) Example traces of confocal time-lapse recordings in single VSN dendritic knobs showing repeatable responses to either CA, to DCA, or to both stimuli. (**b**) Density of knobs that responded exclusively to CA analyzed in control vs. cGαi2$^{-/-}$ VNEs (n = 6, 11, respectively; Mann-Whitney ***, $p < 0.001$). (**c**) Density of knobs that responded exclusively to DCA analyzed in control vs. cGαi2$^{-/-}$ VNEs (n = 6, 11, respectively; Mann-Whitney ***, $p< 0.001$). (**d**) Density of knobs that detected both stimuli analyzed in control vs. cGαi2$^{-/-}$ VNEs (n = 6, 11, respectively; Mann-Whitney **, $p = 0.003$). (**e**) Density of knobs that responded either selectively to CA or DCA or detected both stimuli analyzed in control vs. cGαi2$^{-/-}$ VNEs (n = 6, 11, respectively; Mann-Whitney, * $p < 0.05$; ** $p < 0.01$; *** $p < 0.001$). (**f**) Percentage of all feces-responding knobs that reacted also to CA and/or DCA (n = 15). (**g**) Venn diagram indicating the response overlap between CA and DCA in control VNEs (based on 375 responding knobs).

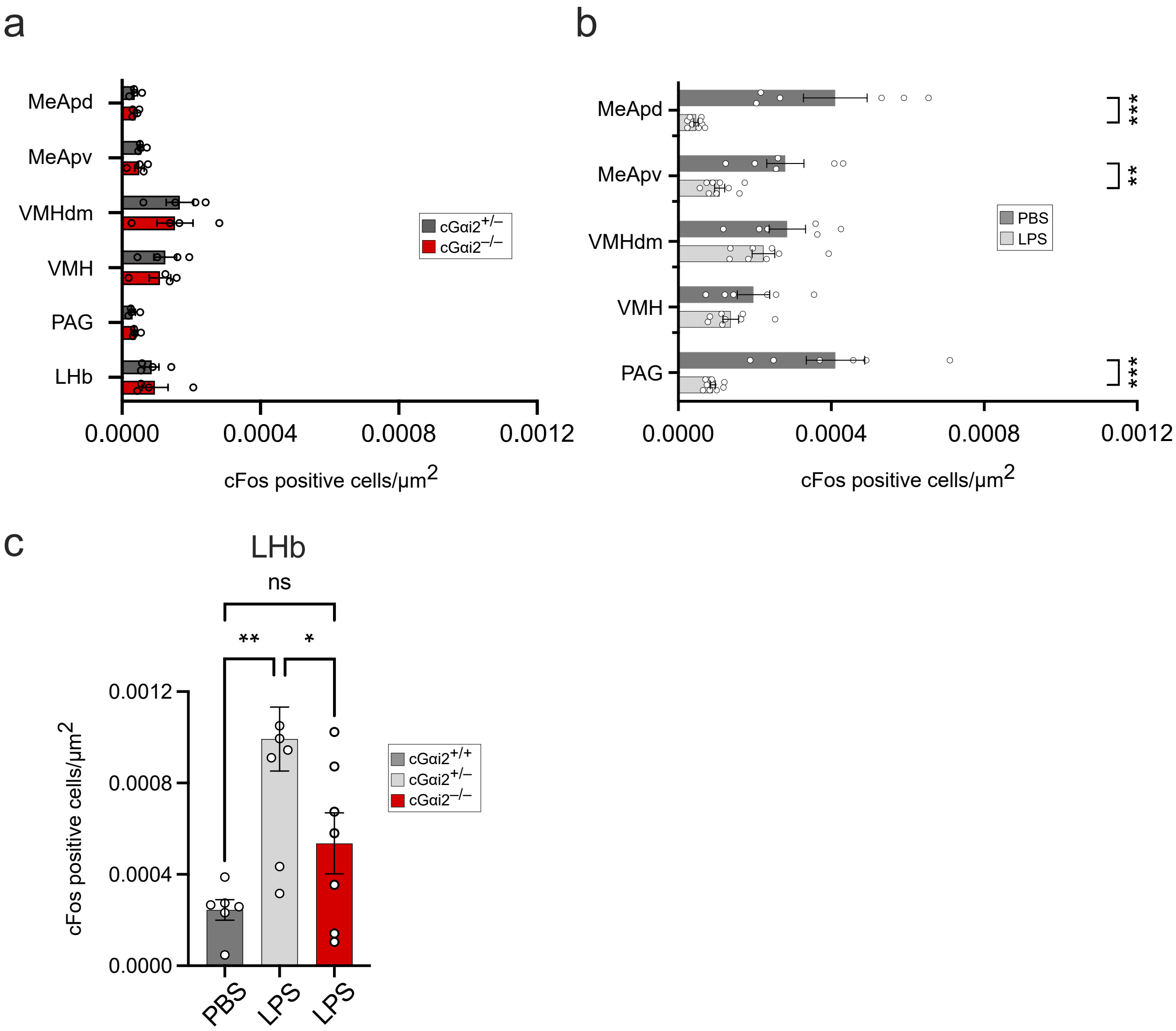

Suppl. Fig. 4. No significant difference in c-Fos activation in brain regions of non-stimulated cGαi2$^{+/–}$ and cGαi2$^{–/–}$ mice. (**a**) Quantification of c-Fos^{+} cells per µm^{2} in MeApd, MeApv, VMHdm, VMH, PAG and LHb of non-stimulated cGαi2$^{+/–}$ and cGαi2$^{–/–}$ mice (n = 4 respectively, Mann-Whitney ns p = 0.20 - >0.99). (**b**) Quantification of c-Fos^{+} cells per mm^{2} in MeApd, MeApv, VMHdm, VMH and PAG of cGαi2$^{+/+}$ and cGαi2$^{+/–}$ mice after exposure to PBS-urine or LPS-urine. Statistically significant differences were observed in the MeApv, MeApd and PAG (n = 6 cGαi2$^{+/+}$ and 6-8 cGαi2$^{+/–}$ mice). No significant differences were observed in the VMH and VMHdm (ns, p = 0.34 and p = 0.41, respectively). Mann-Whitney, **p < 0.01, ***p < 0.001. Open circles represent individual mice. (**c**) Quantification of c-Fos^{+} cells per mm^{2} in LHb of cGαi2$^{+/+}$, cGαi2$^{+/–}$ and cGαi2$^{–/–}$ mice after exposure to PBS-urine or LPS-urine. cGαi2$^{+/+}$ mice exposed to PBS-urine and cGαi2$^{–/–}$ mice exposed to LPS-urine show a statistically significant reduction of their c-Fos activation compared to cGαi2$^{+/–}$ mice exposed to LPS-urine (n = 6 cGαi2$^{+/+}$, n= 8 cGαi2$^{+/–}$ and n = 7 cGαi2$^{–/–}$; one-way ANOVA $F(2, 19)$ = 9.104 p = 0.0017 followed by Holm-Šídák's post-hoc test *p < 0.05, **p < 0.01). No significant differences were observed between cGαi2$^{+/+}$ mice exposed to PBS-urine and cGαi2$^{–/–}$ mice exposed to LPS-urine (ns, Holm-Šídák's post-hoc test p = 0.14).
